# Association between obesity and neurological outcomes among out-of-hospital cardiac arrest patients: The SOS-KANTO 2017 study

**DOI:** 10.1016/j.resplu.2023.100513

**Published:** 2023-11-23

**Authors:** Makoto Aoki, Shotaro Aso, Masaru Suzuki, Takashi Tagami, Yusuke Sawada, Hideo Yasunaga, Nobuya Kitamura, Kiyohiro Oshima, behalf of the SOS-KANTO 2017 Study Group

**Affiliations:** a Advanced Medical Emergency Department and Critical Care Center, Japan Red Cross Maebashi Hospital, Gunma, Japan; b Department of Real World Evidence, Graduate School of Medicine, the University of Tokyo, Tokyo, Japan; c Department of Emergency Medicine, Tokyo Dental College of Ichikawa General Hospital, Chiba, Japan; d Department of Emergency and Critical Care Medicine, Nippon Medical School Musashikosugi Hospital, Kanagawa, Japan; e Department of Emergency Medicine, Gunma University Graduate School of Medicine, Gunma, Japan; f Department of Biostatistics and Bioinformatics, Graduate School of Medicine, the University of Tokyo, Tokyo, Japan; g Department of Emergency and Critical Care Medicine, Kimitsu Chuo Hospital, Chiba Japan

**Keywords:** Obesity, Body mass index, Out-of-hospital cardiac arrest, Outcome

## Abstract

**Aim:**

To assess the association between body mass index (BMI) and neurological outcomes among patients with out-of-hospital cardiac arrest (OHCA).

**Methods:**

This prospective, multicenter, observational study conducted between 2019 and 2021 included adults with OHCA who were hospitalized after return of spontaneous circulation. Based on the BMI, the patients were categorized as underweight (BMI < 18.5 kg/m^2^), normal weight (BMI 18.5–24.9 kg/m^2^), overweight (BMI 25.0–29.9 kg/m^2^), or obese (BMI ≥ 30 kg/m^2^). The normal weight group served as the reference. Favorable neurological outcomes were defined as a Cerebral Performance Category score of ≤2 at 30 days. Multivariate logistic regression analyses were performed to adjust for patient characteristics, OHCA circumstances, and time variables.

**Results:**

Of the 9,909 patients with OHCA who presented during the study period, 637 were eligible, of whom 10.8% (69/637), 48.9% (312/637), 27.6% (176/637), and 12.5% (80/637) were underweight, normal weight, overweight, and obese, respectively. These groups had favorable neurological outcome in 23.2%, 29.2%, 20.5%, and 16.2% of patients, respectively. Obese and overweight patients had a significantly lower rate of favorable neurologic outcomes (adjusted odds ratio [OR] = 0.35; 95% confidence interval [CI] = 0.16–0.77; adjusted OR = 0.53; 95% CI = 0.31–0.90, respectively) than those with a normal weight.

**Conclusions:**

Obese and overweight patients with OHCA have reduced rates of favorable neurological outcomes, suggesting that clinicians should pay attention to the BMI of patients.

## Background

Obesity is a major risk factor for numerous chronic conditions, such as cardiovascular disease, diabetes mellitus, cancers, and musculoskeletal disorders, and an increased risk of mortality.^1^, ^2^, ^3^ Furthermore, obesity is associated with an increased risk of cardiac arrest after adjusting for other risk factors.^4^ Obese patients account for 300,000 cases of sudden cardiac arrest in the United States annually.^5^ Therefore, the association between obesity and outcomes among patients with out-of-hospital cardiac arrest (OHCA) has gained scientific attention.

The obesity paradox theory suggests that obesity is associated with a lower mortality compared to a normal weight or underweight. This theory was reported in the early 1990s for patients with heart failure,^6^, ^7^ but was later extended to other cardiovascular conditions, such as coronary artery disease,^8^ stroke,^9^ and hypertension,^10^ as well as non-cardiovascular diseases.^11^, ^12^, ^13^

Our previous report showed that obesity was associated with unfavorable neurological outcomes.^14^ However, other studies have shown no association between obesity and neurological outcomes among patients with OHCA.^15^, ^16^ Few studies have investigated the association between obesity and OHCA outcomes. Therefore, it remains unknown whether obesity is associated with unfavorable neurological outcomes in patients with OHCA.

Our study aimed to determine the association between body mass index (BMI) and neurological outcomes among patients with OHCA using prospective observational data. We hypothesized that resuscitation is difficult for obese patients, leading to an association between obesity and unfavorable neurological outcomes.

## Methods

The SOS-KANTO 2017 study was a prospective, multicenter (42 emergency hospitals), observational study conducted between September 2019 and March 2021 among patients with OHCA from the Kanto area of Japan. The SOS-KANTO study group has investigated multiple clinical issues related to OHCA since 2002 and regularly performs prospective observational studies with preregistered research hypotheses. The institutional review board of each participating hospital approved the study protocol, including the institutional review board of Gunma University Hospital (HS2019-004). The requirement of informed consent was waived because of the anonymous nature of the data used.

### Study setting and design

Cardiac arrest was defined as the cessation of mechanical heart activity confirmed by the absence of signs of circulation. Adult OHCA patients (aged 16–64 years) who were hospitalized after resuscitation were included. We excluded patients with moderate to severe clinical frailty or traumatic cardiac arrest.

### Variables collected for analysis

Out-of-hospital information regarding OHCA was prospectively collected by emergency medical service providers in the standard Utstein-style template. In-hospital information was collected by the treating physicians at each institution. The collected data included age, sex, height, weight, clinical frailty scale, comorbidities for the Charlson Comorbidity Index; out-of-hospital information, including place of cardiac arrest, witness status, presence of bystander, initial cardiac rhythm on emergency medical service arrival; in-hospital information, including date of admission and discharge, therapeutic measures and laboratories; time variables, including time of witness of cardiac arrest, emergency call, cardiopulmonary resuscitation (CPR) initiation, and return of spontaneous circulation (ROSC); and cause of cardiac arrest that was determined by treating physicians. The height and body weight of patients were measured upon hospital admission following each hospital’s protocol, and uniform measurement standards were not established. The survival status and Pittsburgh Cerebral Performance Categories for neurological function were available at hospital discharge and 30 days after admission. We classified BMI according to the WHO criteria (underweight, BMI < 18.5 kg/m^2^; normal weight, BMI 18.5–24.9 kg/m^2^; overweight, BMI 25.0–29.9 kg/m^2^; obese, BMI ≥ 30 kg/m^2^), with normal-weight patients serving as the reference group.^17^ Mild frailty was defined as mildy frail in clinical frailty scale. No-flow time was defined as the interval between the witness of cardiac arrest and CPR initiation. In cases of unavailable witness, no-flow time was defined as the interval between the time of the emergency call and the time of cardiac arrest. Low-flow time was defined as the interval between CPR initiation and ROSC. The time of ROSC was recorded before and after hospital arrival.

### Outcomes

The primary outcome was favorlable neurological outcome at 30 days, defined as Cerebral Performance Categories 1 (good recovery) and 2 (moderate disability).^18^ The secondary outcome was survival at 30 days.

### Statistical analysis

Continuous variables were presented as median and interquartile range (IQR), and categorical variables were presented as percentages and numbers. Differences between the BMI groups were analyzed with a Kruskal–Wallis H-test for continuous variables. Categorical variables were compared using the χ^2^ test.

Missing data were replaced with a set of substituted plausible values by creating 20 completely filled datasets using a Markov chain Monte Carlo algorithm, known as chained equations imputation.^19^

Multivariate logistic regression analyses were performed to assess the association between BMI and outcomes, with the BMI category as the categorical variable and all available possible confounders: age, sex, mild frailty, Charlson Comorbidity Index, presence of witnesses or bystanders, initial shockable rhytm, presumed cardiogenic cause of cardiac arrest, and low-flow time.

All analyses were performed using R software (version 4.2.2; R Foundation for Statistical Computing, Vienna, Austria). P-value < 0.05 was considered statistically significant.

## Results

**Fig. 1:**
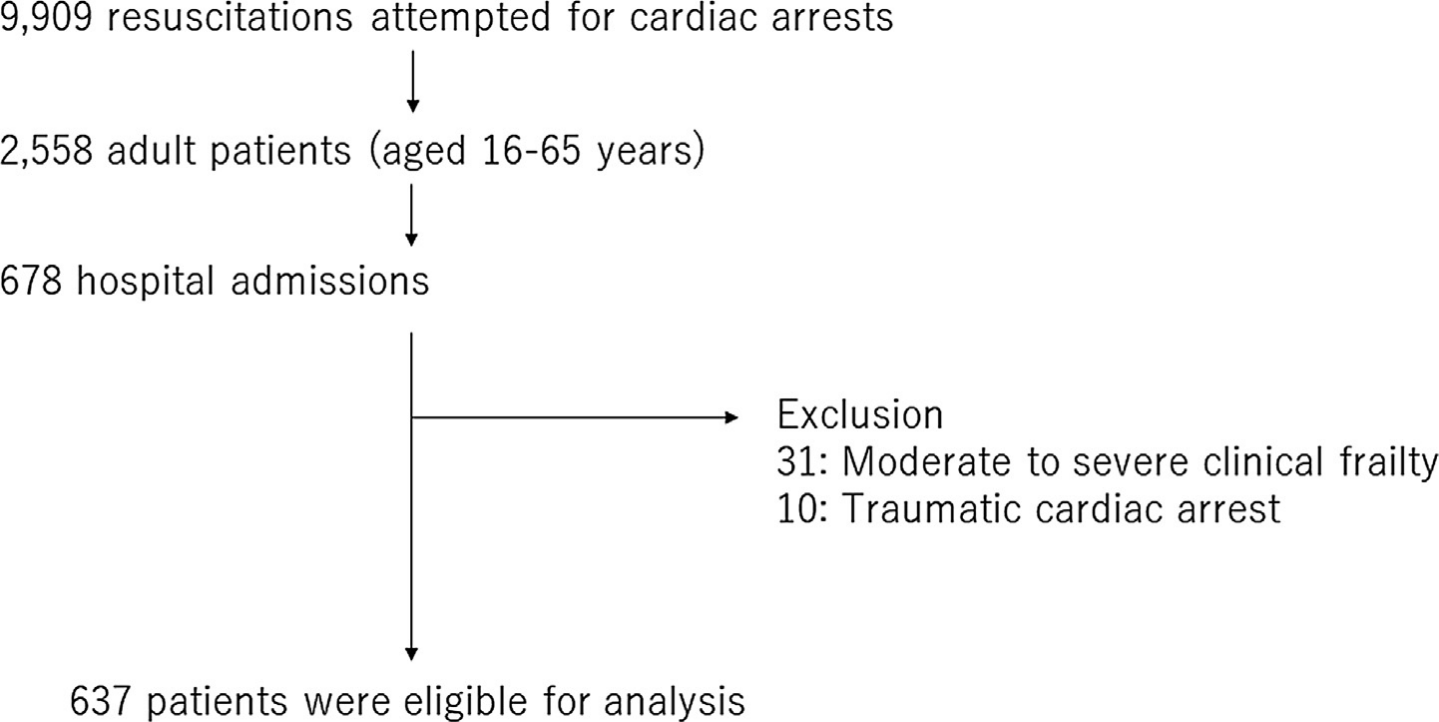
Flow diagram of patients. BMI, body mass index.

A flow diagram of patients included in this study is shown in Fig. 1. Of a total of 9,909 OHCA patients with OHCA, 678 were hospitalized. After the exclusion of 41 patients, 637 were eligible. The proportions of missing BMI information and favorable neurological outcomes at 30 days were 31.0% and 5.9%, respectively. According to the WHO criteria, the patients were classified as underweight (10.8%), normal weight (48.9%), overweight (27.6%), and obesity (12.5%).

Table 1 showed the baseline characteristics of patients based on the BMI groups. The proportions of witnesses, initial shockable rhythm, and presumed cardiogenic cause of cardiac arrest in the underweight group were lower than those in other groups. Regarding therapeutic measures, the obese group received a higher dose of adrenaline and more frequently underwent extracorporeal cardiopulmonary resuscitation. The PaO2 values at the time of ROSC were lower in the obese group than in other groups.

**Table 1:** Patient characteristics according to BMI category.

Variable	BMI < 18.5	BMI 18.5–24.9	BMI 25.0–30.0	BMI > 30.0	p-value
	*n* = 69	*n* = 312	*n* = 176	*n* = 80	
Demographics					
Age	53 (40–59)	52 (43–59)	52 (45–57)	52 (47–58)	0.85
Sex (male), n (%)	44 (63.8)	232 (74.4)	136 (77.3)	62 (77.5)	0.154
Frailty scale, n (%)					
Mild Frailty	11 (15.9)	33 (10.6)	23 (13.1)	8 (10.0)	0.55
Charlson Comorbidity Index, median (IQR)	0 (0–1)	0 (0–1)	0 (0–1)	0 (0–3)	0.233
Circumstances, n (%)					
Witness	34 (49.3)	219 (70.2)	133 (75.6)	65 (81.2)	< 0.001
Bystander	34 (49.3)	177 (56.7)	97 (55.1)	49 (61.3)	0.515
Initial shockable rhythm	14 (20.3)	113 (36.2)	69 (39.2)	20 (25.0)	0.009
Presumed cardiogenic cause of cardiac arrest	29 (42.0)	196 (62.8)	114 (64.8)	55 (68.8)	0.003
No-flow time, min (IQR)	4 (0–9)	3 (0–8)	4 (0–9)	3 (0–9)	0.495
Low-flow time, min (IQR)	28 (16–43)	29 (19–41)	29 (17–44)	34 (18–52)	0.356
Therapeutic measures					
Adrenaline dose, mg	0 (0–2)	1 (0–2)	2 (0–3)	2 (0–4)	< 0.001
ECPR, n (%)	10 (14.5)	80 (25.6)	56 (31.8)	35 (43.8)	< 0.001
PCI, n (%)	7 (10.1)	67 (21.5)	44 (25.0)	21 (26.2)	0.058
TTM, n (%)	28 (40.6)	146 (46.8)	88 (50.0)	36 (45.0)	0.593
Laboratory parameters at ROSC, median					
PaCO_2_, mmHg	51 (36–71)	45 (35–69)	48 (35–72)	51 (39–83)	0.187
PaO_2_, mmHg	216 (84–461)	180 (84–343)	149 (78–282)	108 (70–215)	0.002
Base excess, mEq/L	−12 (−18 to −7)	−12 (−19 to −7)	−14 (−20 to −7)	−15 (−21 to −9)	0.372
Lactate, mmol/L	10.4 (7.0–15.0)	10.5 (7.0–14.6)	10.0 (6.7–13.7)	9.4 (5.7–13.6)	0.48

BMI, body mass index; ROSC, return of spontaneous circulation; EMS, emergency medical service; IQR, interquartile range; ECPR, extracorporeal cardiopulmonary resuscitation; PCI, percutaneous coronary intervention; TTM, targeted temperature management.

## Favorable neurological outcomes

The rate of favorable neurological outcomes was 24.4% (156/637). Table 2 shows the univariate analysis of outcomes between BMI groups. The proportions of favorable neurological outcomes at 30 days were 23.2% (16/69), 29.2% (91/312), 20.5% (36/176), and 13/80 (16.2%) for the underweight, normal weight, overweight, and obesity groups, respectively. The proportion of favorable neurological outcome was lower in the obese group than the other groups. Table 3 presents the results of the multivariable logistic regression model for favorable neurological outcomes. Obesity and overweight were significantly associated with reduced favorable neurological outcomes at 30 days compared to normal weight (adjusted odds ratio [OR] = 0.35, 95% confidence interval [CI] = 0.16–0.77; adjusted OR = 0.53, 95% CI = 0.31–0.90, respectively). Underweight was not associated with favorable neurological outcomes at 30 days compared to normal weight.

**Table 2:** Favorable neurological outcomes and survival at 30 days.

Variable	BMI < 18.5	BMI 18.5–24.9	BMI 25.0–30.0	BMI > 30.0	p-value
	*n* = 69	*n* = 312	*n* = 176	*n* = 80	
Favorable neurological outcome, n (%)	16 (23.2)	91 (29.2)	36 (20.5)	13 (16.2)	0.041
Survival at 30 days, n (%)	28 (40.6)	131 (42.0)	67 (38.1)	21 (26.2)	0.079

BMI, body mass index.

**Table 3:** Multivariable logistic regression with multiple imputation for analysis of favorable neurological outcomes and survival at 30 days.

Variable	BMI < 18.5	BMI 18.5–24.9	BMI 25.0–30.0	BMI > 30.0
	*n* = 69	*n* = 312	*n* = 176	*n* = 80
Favorable neurological outcome, AOR (95% CI)	1.33 (0.61–2.87)	Reference	0.53 (0.31–0.90)	0.35 (0.16–0.77)
Survival at 30 days, AOR (95% CI)	1.85 (0.93–3.66)	Reference	0.81 (0.51–1.30)	0.41 (0.21–0.82)

BMI, body mass index; AOR, adjusted odds ratio; CI, confidence interval.

Adjusted variables were age, sex, mild frailty, Charlson Comorbidity Index, presence of witnesses, presence of bystanders, initial shockable rhythm, presumed cardiogenic cause of cardiac arrest and low-flow time.

## Survival at 30 days

The overall proportions of survival at 30 days were 38.7% (247/637). The proportions of survival at 30 days were 40.6% (28/69), 42.0% (131/312), 38.1% (67/176), and 26.3% (21/80) in underweight, normal weight, overweight, and obesity groups, respectively (Table 2). Obesity was significantly associated with reduced survival at 30 days compared to normal weight (adjusted OR = 0.41, 95% CI = 0.21–0.82). Underweight and overweight were not associated with survival at 30 days compared to normal weight.

## Discussion

This study evaluated the association between BMI and outcomes among patients with OHCA. Multivariable analyses showed that the WHO-defined obese and overweight groups were associated with reduced favorable neurological outcome and survival at 30 days compared to the normal weight group.

Resuscitation for a patient with a higher BMI is challenging, and CPR guidelines focused on this population have been proposed.^20^ A recent paper from Kosmopoulos et al. demonstrated that obese patients with OHCA (BMI > 30 kg/m^2^) had a longer resuscitation time, higher requirements for mechanical support, and lower proportions of survival to hospital discharge compared with those who have a BMI ≤30 kg/m.^21^ Our results are similar to those of a previous report. Impaired chest compression mechanics have been demonstrated on a simulation manikin, and the chest compression depth was significantly lower on the obese manikin.^22^ Thoracic impendence increases and the proportion of defibrillation success was decreased with the BMI.^23^ Additionally, our results suggest that decreased PaO2 level was associated with increased BMI. Chest wall elastance and wall compliance may also be affected by obesity.^24^

A recent systematic review and meta-analysis revealed that underweight was associated with reduced favorable neurological outcomes.^25^ This was explained by malnutrition, baseline comorbidities, and a lower rate of good prognostic factors, such as shockable rhythm.^25^ Similarly, the lower proportions of witnesses, bystanders, initial shockable rhythm, and presumed cardiogenic cause in underweight patients was not associated with worse outcomes. The possible reason is that we adjusted for the clinical frailty scale. The association between weight loss and functional decline has been reported,^26^ and clinical frailty is associated with the mortality among patients with OHCA.^27^

The clinical implication of this study is that resuscitation strategy can be decided and outcomes for patients with OHCA can be predicted using the patients’ BMI. Although the BMI of patients with OHCA is often not available in the emergency setting, we can predict the difficulty level of the resuscitation by inspecting the patients’ body type and predict the outcomes before the BMI is available.

We acknowledge that the current study has several limitations. First, although obesity was associated with reduced favorable neurological outcomes, causality cannot be inferred because this study had an observational design. However, randomized controlled trials to assess the association between BMI and outcomes among patients with OHCA may not be feasible. Second, BMI had the inherent limitations as a measure of obesity as it does not account for variation in body composition and body fat distribution is more predictive of all cause mortality,^28^ while, BMI is a relatively simple and low cost indirect measure for assessing obesity, and we used the WHO BMI classification. Third, we included only patients who achieved ROSC. Therefore, the proportions of favorable neurological and survival outcomes were higher than those in previous studies.^25^ The results in this study were not able to be applied to OHCA patients who did not achieve ROSC. Fourth, we assessed the association between obesity and neurological outcomes among OHCA. Nevertheless, there are still unanswered questions, such as the higher prevalences of witnesses and bystander, to better understand the implication of obesity in OHCA. However, we conducted multivariable logistic regression adjusting for these variables.

## Conclusions

Obesity and overweight are associated with reduced favorable neurological outcomes. We can determine the optimal resuscitative strategy and prognosis by considering the BMI of patients with OHCA.

## Funding

No funding.

## CRediT authorship contribution statement

**Makoto Aoki:** Conceptualization, Methodology, Formal analysis, Investigation, Writing – original draft. **Shotaro Aso:** Methodology, Formal analysis, Investigation, Writing – original draft, Writing – review & editing. **Masaru Suzuki:** Writing – review & editing, Supervision. **Takashi Tagami:** Writing – review & editing, Project administration. **Yusuke Sawada:** Writing – review & editing, Visualization. **Hideo Yasunaga:** Methodology, Formal analysis, Writing – review & editing. **Nobuya Kitamura:** Writing – review & editing, Project administration. **Kiyohiro Oshima:** Writing – review & editing, Visualization, Supervision.

## Declaration of competing interest

The authors declare that they have no known competing financial interests or personal relationships that could have appeared to influence the work reported in this paper.
